# Pulmonary artery diameters, cross sectional areas and area changes measured by cine cardiovascular magnetic resonance in healthy volunteers

**DOI:** 10.1186/s12968-016-0230-9

**Published:** 2016-03-03

**Authors:** Elisabeth D Burman, Jennifer Keegan, Philip J Kilner

**Affiliations:** CMR Unit, Royal Brompton Hospital, Sydney Street, SW3 6NP London, UK

**Keywords:** Pulmonary arteries, MRI, Pulmonary hypertension, Congenital heart disease

## Abstract

**Background:**

We measured by cine cardiovascular magnetic resonance (CMR) main and branch pulmonary artery diameters and cross sectional areas in diastole and systole in order to establish normal ranges and the effects on them of age, gender and body surface area (BSA). Documentation of normal ranges provides a reference for research and clinical investigation in the fields of congenital heart disease, pulmonary hypertension and connective tissue disorders.

**Methods:**

We recruited 120 healthy volunteers: ten males (M) and ten females (F) in each decile between 20 and 79 years, imaging them in a 1.5 Tesla CMR system. Scout acquisitions guided the placement of steady state free precession cine acquisitions transecting the main, right and left pulmonary arteries (MPA, RPA and LPA). Cross sections were rarely quite circular.

**Results:**

From all subjects, the means of the greater and lesser orthogonal diastolic diameters in mm were: MPA, 22.9 ± 2.4 (M) and 21.2 ± 2.1 (F), RPA 16.6 ± 2.8 (M) and 14.7 ± 2.2 (F), and LPA 17.3 ± 2.5 (M) and 15.9 ± 2.0 (F), p < 0.0001 between genders in each case. The diastolic diameters increased with BSA and age, and plots are provided for reference. From measurements of minimum diastolic and maximum systolic cross sectional areas, the % systolic distensions were: MPA 42.7 ± 17.2 (M) and 41.8 ± 15.7 (F), RPA 50.6 ± 16.9 (M) and 48.2 ± 14.5 (F), LPA 35.6 ± 10.1 (M) and 35.2 ± 10.3 (F), and there was a decrease in distension with age (p < 0.0001 for the MPA).

**Conclusions:**

Measurements of MPA, RPA and LPA by cine CMR are provided for reference, with documentation of their changes with age and BSA.

## Background

Cardiovascular magnetic resonance (CMR) allows non-invasive visualisation of the main, right and left pulmonary arteries (MPA, RPA and LPA) without exposure to ionising radiation. Measurements of normal dimensional ranges are fundamental to the identification of abnormal size in pathologies such as pulmonary hypertension [1, 2] connective tissue disorders,[3] congenital heart disease [4] and acquired cardio-pulmonary diseases [5]. CMR cine imaging has been found to be advantageous for this purpose because of its freedom from contrast administration and its ability to measure both systolic and diastolic dimensions of the compliant vessels [4]. However, in a recent review that collected suitably documented reports of various normal values measured by CMR, pulmonary arterial measurements were missing [6]. This may in part have been due to the lack of any previous study where appropriately standardized methods of CMR acquisition and measurement were obtained in sufficient numbers of volunteers to provide ranges of normal values with respect to gender and age. Here we report measurements of cross sectional areas, systolic change of area (distension) and the diameters of pulmonary arteries from prospectively acquired CMR cine acquisitions in healthy volunteers. Our aim was to establish normal reference values and to document any changes in them with age and body surface area, and differences between genders.

## Methods

### Study cohort

One hundred and twenty normotensive volunteers were recruited, 10 of each gender in each decile between 20 and 80 years. Ethical approval had been obtained from the local regional ethics committee, and informed consent was obtained from all subjects. Height, weight, and blood pressure were measured (Table 1). BSA was calculated according to the Du Bois formula [7]. Individuals with known coronary heart disease, valvular disease, heart failure, hypertension (systolic blood pressure >150 mm Hg, diastolic blood pressure of >90 mm Hg as measured by mercury sphygmomanometer), or atrial fibrillation were excluded.

**Table 1:** Characteristics of subjects

Characteristic	Men (*n* = 60)	Women (*n* = 60)
Age	49.4 ± 17.2	49.3 ± 16.7
Weight (kg)	80.2 ± 12.9	64.1 ± 9.3
Height (m)	1.8 ± 0.1	1.6 ± 0.1
Body surface area (m^2^)	2.0 ± 0.2	1.7 ± 0.1
Systolic blood pressure (mmHg)	126.4 ± 13.8	123 ± 15.2
Diastolic blood pressure (mmHg)	79.8 ± 8.6	79.6 ± 8.5

Mean ± standard deviation

### Cardiovascular magnetic resonance

All subjects were imaged using a 1.5 Tesla system (Siemens Sonata, Erlangen, Germany). Transaxial and coronal half-fourier acquisition single-shot turbo spin echo (HASTE) multislice pilot images were acquired in diastole during held expiration. Prospectively gated balanced steady-state free precession (bSSFP) cine acquisitions were then acquired, also during held expiration, using the following parameters: echo time of 1.55 ms, 15 views per data segment, temporal resolution of 46 ms without view sharing. Voxel size was 6 × 1.4 × 1.6 mm.

**Fig. 1:**
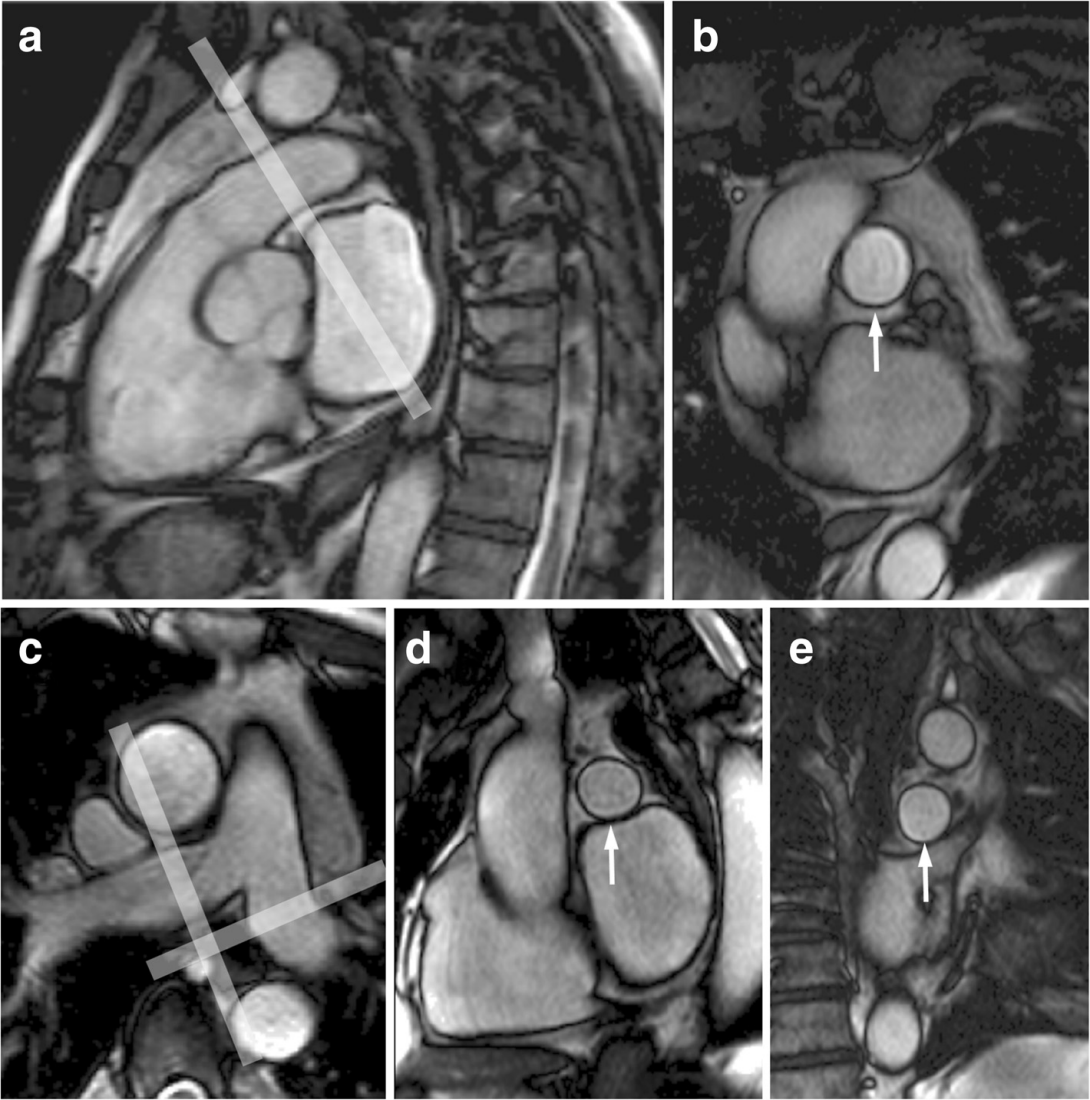
Steady state free precession images of the pulmonary arteries. The main pulmonary artery can be seen in the oblique sagittal image (panel **a**). The pale band shows the location of the cine acquisition that transects it (panel **b**, arrow). The right and left pulmonary arteries can be seen in the scout image (panel **c**). The bands indicate the locations of cine acquisitions transecting the right (panel **d**, arrow) and the left pulmonary artery (panel **e**, arrow)

Cine planes were acquired as follows: an oblique sagittal SSFP cine, located from the transverse multislice scouts to be aligned with the right ventricular outflow tract (RVOT) and pulmonary trunk (Fig. 1a). An oblique cine acquisition was then located to transect the MPA immediately distal to its sino-tubular junction to obtain a cross-section of the MPA (Fig. 1b). Two further cines were then aligned to transect the RPA and LPA, each located across tubular regions about 1 cm distal to the MPA bifurcation as identified on transaxial scout images (Fig. 1c, d and e).

### Measurements of pulmonary arterial area and diameter

The three trans-arterial cines were used to measure the cross sectional areas and diameters of the relatively bright luminal blood signal in the pulmonary arteries at the phases of minimal diastolic and maximal systolic expansion. Hence % systolic distension [(maximum area - minimum area) × 100 ÷ minimum area] of each artery was calculated. As the cross sections were rarely perfectly circular, two diameters were measured at each phase: the greater diameter and the lesser diameter, orthogonal to the greater. The mean of both could then also be used for analysis. The measurements were performed using CMRtools (Cardiovascular Imaging Solutions, London, UK) to manually define lines and area boundaries. Images were loaded in DICOM 3.0 format to the software in which pixel dimensions provided the basis for calculations of length and area, the latter using the rasterization of contours onto a sub pixel grid, the technique having been verified during the development of the software using magnetic resonance images of a calibration phantom.

### Statistical analysis

The means and standard deviations of all area and diameter measurements were calculated for each gender and for each decile. Pulmonary artery distension, area and diameter measurements were plotted with respect to age and the data examined for normality and the presence of outliers. The measurements of any outliers were checked to exclude input errors.

Stepwise backwards multivariate linear regression analysis using the Statistical Package for the Social Sciences (SPSS) Version 10 for Windows, was performed for all pulmonary artery diameters for both sexes with age and BSA as input variables. In each analysis, R^2^ values were determined to give the proportion of the variability in the pulmonary vessel measurements attributable to the regression model.

Following normality checks, unpaired t-tests were performed to compare pulmonary artery distension and all diameters between males and females.

## Results

### Overview of data in tables and figures

**Fig. 2:**
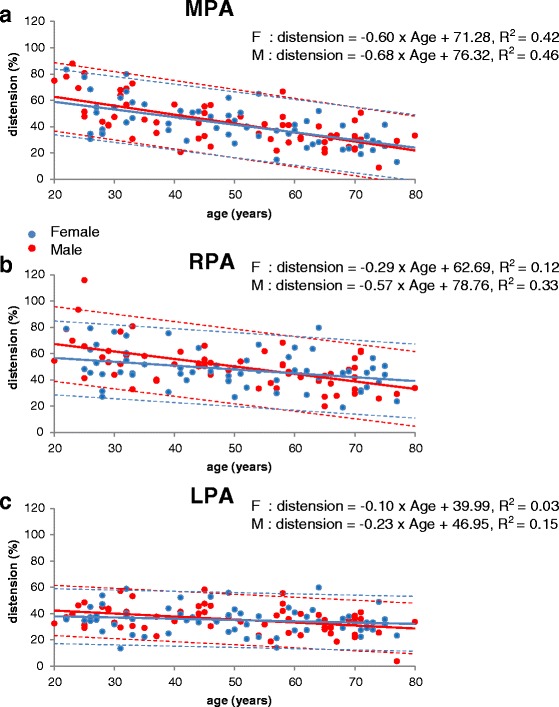
**a**. Pulmonary artery distension (%) of the MPA plotted for all volunteers against age. **b**. Pulmonary artery distension of the RPA plotted against age. **c**. Pulmonary artery distension of the LPA plotted against age. The dotted lines show 95 % prediction intervals.

**Fig. 3:**
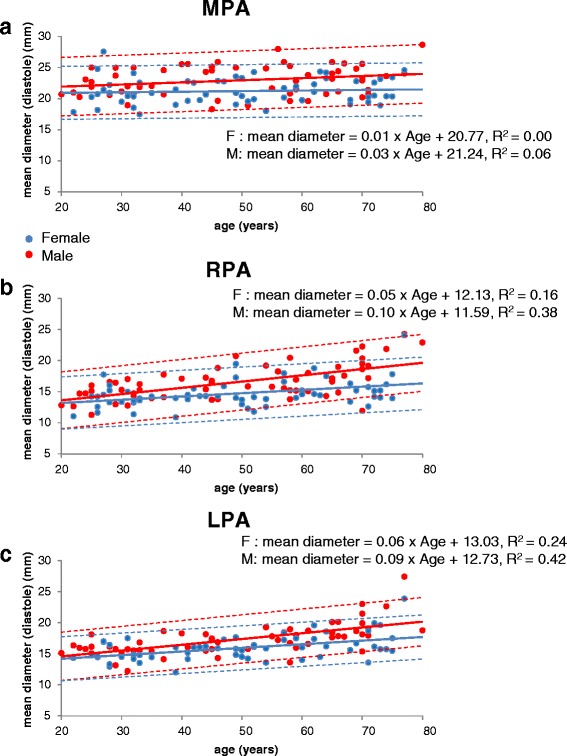
**a**. Pulmonary artery mean diastolic diameter (mm) of the MPA plotted for all volunteers against age. **b**. Pulmonary artery mean diastolic diameter of the RPA plotted against age. **c**. Pulmonary artery mean diastolic diameter of the LPA plotted against age. The dotted lines show 95% prediction intervals

**Fig. 4:**
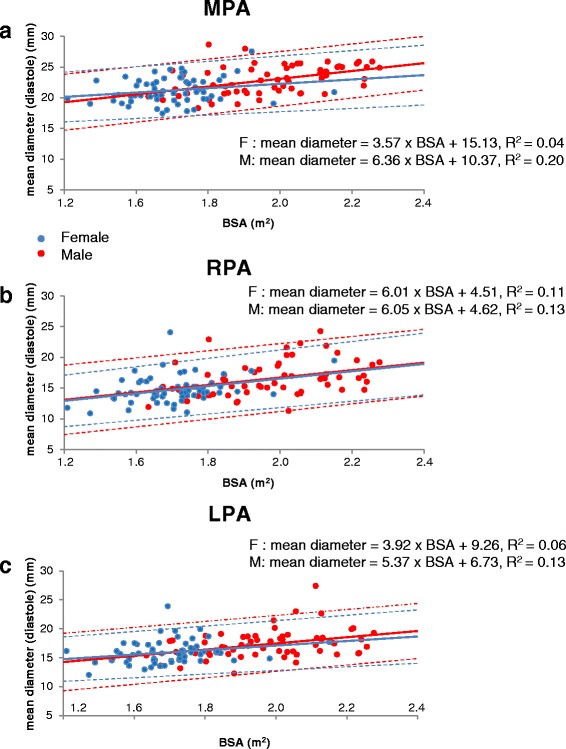
**a**. Pulmonary artery mean diastolic diameter of the MPA plotted in all volunteers against BSA. **b**. Pulmonary artery mean diastolic diameter of the RPA plotted against BSA. **c**. Pulmonary artery mean diastolic diameter of the LPA plotted against BSA. The dotted lines show 95 % prediction intervals. The dotted lines show 95 % prediction intervals

Table 1 shows the body metrics of the male and female cohorts. All pulmonary artery variables analyzed were found to be approximately normally distributed. The measurements of systolic and diastolic areas, systolic and diastolic mean diameters and % systolic distension of the main, right and left pulmonary arteries are shown in Table 2 as means and standard deviations. Measurements of % systolic distension are plotted against age in Fig. 2 together with the 95 % prediction intervals. Plots of mean diastolic vessel diameters against age and against BSA are shown in Figs. 3 and 4, respectively, showing that each measurement tended to increase with age and BSA. Table 3 summarizes multivariate regression analyses for distension, systolic and diastolic cross-sectional areas and systolic and diastolic mean diameters. In brief: In all arteries, systolic and diastolic areas and mean diameters were greater in males than females (*p* < 0.001 for all). For all arteries in all subjects, areas and diameters were greater in systole than diastole (*p* < 0.001 for all). R^2^ values for arterial measurements relative to age and BSA were always higher in the male cohort than the female, and R^2^ values for distension were higher in the MPA than the RPA or LPA for both male and female cohorts. Conversely, R^2^ values for linear measurements in the MPA were lower than values for the RPA and LPA. Diastolic measurements generally showed higher R^2^ values than systolic measurements.

**Table 2:** Mean vessel diameter (mm) in systole and diastole together with area measurements (cm^2^) and % distension, of main pulmonary artery (MPA), right pulmonary artery (RPA) and left pulmonary artery (LPA)

Age	Systolic diameter	Diastolic diameter	Systolic area	Diastolic area	% distension	Age	Systolic diameter	Diastolic diameter	Systolic area	Diastolic area	% distension
	Male MPA						Female MPA				
20–29	28.3 ± 1.8	22.4 ± 1.8	6.3 ± 0.8	3.9 ± 0.6	61.5 ± 18.4	20–29	26.4 ± 3.3	21.6 ± 2.9	5.5 ± 1.4	3.7 ± 1.0	50.7 ± 19.6
30–39	27.3 ± 2.7	21.9 ± 1.8	5.8 ± 1.2	3.8 ± 0.6	53.2 ± 14.9	30–39	25.5 ± 2.8	20.4 ± 2.0	5.1 ± 1.1	3.3 ± 0.6	54.8 ± 13.0
40–49	28.2 ± 3.1	23.5 ± 2.8	6.2 ± 1.3	4.4 ± 1.0	41.4 ± 13.5	40–49	26.5 ± 1.6	21.5 ± 1.6	5.4 ± 0.6	3.7 ± 0.5	46.2 ± 9.6
50–59	27.0 ± 2.7	22.9 ± 3.0	5.7 ± 1.2	4.2 ± 1.0	40.0 ± 12.6	50–59	24.4 ± 2.7	20.7 ± 2.1	4.7 ± 0.9	3.4 ± 0.7	38.5 ± 13.4
60–69	26.5 ± 2.2	23.3 ± 2.1	5.5 ± 0.8	4.3 ± 0.7	29.6 ± 5.8	60–69	25.0 ± 2.7	21.6 ± 2.0	5.0 ± 1.0	3.7 ± 0.7	33.9 ± 10.4
70–79	27.1 ± 2.7	23.6 ± 2.6	5.8 ± 1.2	4.4 ± 0.9	30.7 ± 11.6	70–79	24.0 ± 1.9	21.4 ± 1.9	4.6 ± 0.7	3.6 ± 0.6	26.8 ± 7.9
All subjects	27.4 ± 2.6	22.9 ± 2.4	5.9 ± 1.1	4.2 ± 0.8	42.7 ± 17.2	All subjects	25.3 ± 2.6	21.2 ± 2.1	5.0 ± 1.0	3.6 ± 0.7	41.8 ± 15.7
	Male RPA						Female RPA				
20–29	18.6 ± 2.0	14.3 ± 1.8	2.8 ± 0.6	1.7 ± 0.4	69.1 ± 21.9	20–29	17.0 ± 1.5	14.0 ± 2.0	2.3 ± 0.4	1.5 ± 0.3	53.6 ± 18.0
30–39	18.8 ± 2.1	15.2 ± 1.5	2.8 ± 0.6	1.8 ± 0.4	54.2 ± 15.9	30–39	16.5 ± 1.3	13.3 ± 1.3	2.2 ± 0.3	1.4 ± 0.3	55.7 ± 15.5
40–49	20.3 ± 2.4	16.4 ± 2.3	3.3 ± 0.8	2.2 ± 0.6	53.1 ± 6.4	40–49	18.6 ± 2.0	15.3 ± 1.8	2.8 ± 0.6	1.9 ± 0.5	46.1 ± 7.5
50–59	20.2 ± 2.4	16.6 ± 2.1	3.3 ± 0.8	2.2 ± 0.6	48.2 ± 11.6	50–59	16.8 ± 2.2	14.0 ± 1.9	2.3 ± 0.6	1.6 ± 0.4	44.9 ± 12.7
60–69	20.2 ± 2.7	17.1 ± 2.2	3.3 ± 0.9	2.4 ± 0.6	37.2 ± 9.2	60–69	19.0 ± 2.4	15.8 ± 1.7	2.9 ± 0.7	2.0 ± 0.4	48.2 ± 17.6
70–79	23.4 ± 3.6	19.8 ± 3.5	4.4 ± 1.2	3.2 ± 1.0	42.1 ± 14.0	70–79	18.8 ± 3.3	15.9 ± 3.2	2.9 ± 1.1	2.1 ± 0.9	40.4 ± 10.3
All subjects	20.2 ± 2.9	16.6 ± 2.8	3.3 ± 1.0	2.2 ± 0.8	50.6 ± 16.9	All subjects	17.8 ± 2.4	14.7 ± 2.2	2.6 ± 0.7	1.8 ± 0.6	48.2 ± 14.5
	Male LPA						Female LPA				
20–29	18.8 ± 1.6	15.8 ± 1.2	2.8 ± 0.4	2.0 ± 0.3	39.6 ± 6.8	20–29	17.6 ± 1.2	14.9 ± 1.4	2.5 ± 0.3	1.8 ± 0.3	38.7 ± 7.9
30–39	18.2 ± 1.9	15.5 ± 1.7	2.6 ± 0.5	1.9 ± 0.4	37.5 ± 11.0	30–39	16.9 ± 1.3	14.7 ± 1.4	2.3 ± 0.3	1.8 ± 0.3	33.2 ± 13.0
40–49	19.8 ± 1.4	16.7 ± 1.3	3.2 ± 0.5	2.2 ± 0.3	42.2 ± 7.8	40–49	18.7 ± 1.4	15.9 ± 1.1	2.8 ± 0.4	2.0 ± 0.3	40.4 ± 9.9
50–59	20.0 ± 2.0	17.2 ± 1.7	3.2 ± 0.6	2.4 ± 0.5	35.2 ± 11.3	50–59	18.1 ± 1.6	15.9 ± 1.5	2.7 ± 0.5	2.1 ± 0.4	30.3 ± 10.7
60–69	21.0 ± 1.5	18.4 ± 1.1	3.5 ± 0.5	2.7 ± 0.3	30.2 ± 6.9	60–69	19.1 ± 2.1	16.4 ± 1.7	3.0 ± 0.6	2.2 ± 0.5	37.2 ± 9.6
70–79	23.1 ± 2.7	20.4 ± 3.4	4.3 ± 1.0	3.4 ± 1.1	29.2 ± 11.0	70–79	20.1 ± 3.1	17.6 ± 3.0	3.3 ± 1.0	2.5 ± 0.9	31.2 ± 7.5
All subjects	20.1 ± 2.4	17.3 ± 2.5	3.3 ± 0.8	2.4 ± 0.7	35.6 ± 10.1	All subjects	18.4 ± 2.1	15.9 ± 2.0	2.8 ± 0.6	2.1 ± 0.5	35.2 ± 10.3

**Table 3:** A. Summary of multivariate regression analyses of systolic and diastolic areas, systolic and diastolic mean diameters and distension, in the main, right, and left pulmonary arteries of the male cohort. (*ns* = not significant) B. Summary of multivariate regression analyses of systolic and diastolic areas, systolic and diastolic mean diameters and distension, in the main, right, and left pulmonary arteries of the female cohort. (*ns* = not significant)

A. Male							
				BSA		Age	
		R^2^	Constant	Slope	Significance	Slope	significance
MPA	Systolic area	0.14	1.03	2.46	*p*<0.01	-	ns
	Systolic diameter (mean)	0.15	1.58	0.59	*p*<0.01	-	ns
	Diastolic area	0.31	−1.35	2.44	*p*<0.001	0.01	p<0.05
	Diastolic diameter (mean)	0.27	8.30	6.50	*p*<0.001	0.04	p<0.05
	Distension	0.54	133.07	−28.49	*p*<0.01	−0.69	p<0.001
RPA	Systolic area	0.41	−2.52	2.21	*p*<0.001	0.03	p<0.001
	Systolic diameter	0.40	−0.20	0.71	*p*<0.001	0.01	p<0.001
	Diastolic area	0.52	−2.51	1.69	*p*<0.001	0.03	p<0.001
	Diastolic diameter (mean)	0.52	−1.24	6.44	*p*<0.001	0.10	p<0.001
	Distension	0.33	78.60	-	Ns	−0.57	p<0.001
LPA	Systolic area	0.56	−2.15	2.00	*p*<0.001	0.03	p<0.001
	Systolic diameter (mean)	0.54	0.39	0.60	*p*<0.001	0.01	p<0.001
	Diastolic area	0.53	−2.11	1.61	*p*<0.001	0.03	p<0.001
	Diastolic diameter (mean)	0.56	1.32	5.73	*p*<0.001	0.10	p<0.001
	Distension	0.15	46.95	-	ns	−0.23	p<0.01
B. Female							
MPA	Systolic area	0.13	2.34	2.06	*p*<0.05	−0.02	p<0.05
	Systolic diameter (mean)	0.14	1.94	0.48	*p*<0.05	−0.01	p<0.05
	Diastolic area	-	-	-	ns	-	ns
	Diastolic diameter (mean)	-	-	-	ns	-	ns
	Distension	0.40	71.28	-	ns	−0.60	p<0.001
RPA	Systolic area	0.24	−1.38	1.87	*p*<0.01	0.02	p<0.01
	Systolic diameter (mean)	0.24	0.43	0.64	*p*<0.01	0.01	p<0.01
	Diastolic area	0.26	−1.21	1.34	*p*<0.05	0.01	p<0.05
	Diastolic diameter (mean)	0.27	1.96	5.99	p<0.01	0.05	p<0.001
	Distension	0.12	62.7	-	ns	−0.30	p<0.01
LPA	Systolic area	0.30	−0.70	1.51	*p*<0.05	0.18	p<0.001
	Systolic diameter (mean)	0.32	0.69	0.50	*p*<0.01	0.01	p<0.001
	Diastolic area	0.27	−0.25	-	ns	0.02	p<0.001
	Diastolic diameter (mean)	0.30	6.426	3.9	*p*<0.05	0.06	p<0.001
	Distension	-	-	-	ns	-	ns

### Pulmonary artery distension

#### Main pulmonary artery

Stepwise backwards multivariate linear regression analysis identified age as an independent predictor of MPA distension in the male and female cohorts (*p* < 0.001 for both). In the male cohort, BSA was also an independent predictor (p < 0.01) but this was not the case in the female cohort. R^2^ values were higher in the male cohort than in the female cohort (R^2^ = 0.54 versus R^2^ = 0.40). There was no significant difference between male and female distension.

### Right pulmonary artery

In the right pulmonary artery, stepwise multivariate regression analysis selected age as an independent predictor in both males (*p* < 0.001)) and females (p < 0.01). BSA was not an independent predictor in either cohort. R^2^ values were again higher in the male cohort than in the female cohort (R^2^ = 0.33 versus R^2^ = 0.12) respectively. There was no significant difference between male and female distension.

### Left pulmonary artery

Age was an independent predictor of left pulmonary artery distension in the male cohort (*p* < 0.01) but not in the female cohort. BSA was not an independent predictor in either cohort. R^2^ was 0.15 for the male subjects. There was no significant difference between male and female distension.

### Linear measurements of the pulmonary arteries

#### Main pulmonary artery

Stepwise regression selected BSA as an independent predictor of systolic and diastolic mean diameters in the male cohort (*p* < 0.01 and *p* < 0.001 respectively). In addition, age was an independent predictor of diastolic mean diameter (*p* < 0.05). R^2^ values were 0.15 and 0.27 for the systolic and diastolic mean diameters respectively. For the female cohort, age and BSA were independent predictors of mean systolic diameter (both *p* < 0.05) but the R^2^ value was low (0.14). Neither age nor BSA were predictors of mean diastolic diameter in this cohort.

### Right pulmonary artery

For the male cohort, both age and BSA were independent predictors mean systolic and mean diastolic diameters (all *p* < 0.001). R^2^ values for systolic and diastolic measurements were 0.40 and 0.52 respectively. For the female cohort, age was an independent predictor of mean systolic diameter (*p* < 0.01) and mean diastolic diameter (*p* < 0.001). BSA was also a predictor of both (*p* < 0.01). R^2^ values for systolic and diastolic measurements were 0.24 and 0.27 respectively.

### Left pulmonary artery

Age was an independent predictor of mean systolic and mean diastolic diameters in both male and female cohorts (all p < 0.001). BSA was also a predictor of both systolic and diastolic diameters in both cohorts (p < 0.001 for systolic and diastolic diameters in males, p < 0.01 for systolic diameter in females, *p* < 0.05 for diastolic diameter in females). For males, R^2^ values for systolic and diastolic mean diameters were 0.54 and 0.56 respectively. For females, the corresponding R^2^ values were 0.32 and 0.30.

### Effect of age on pulmonary artery distension and linear measurements

Figure 2 shows a plot of distension against age in the MPA, RPA and LPA for both males and females. For males, distension fell by 7 % per decade, 6 % per decade and 2 % per decade for the MPA, RPA and LPA respectively. For females, the corresponding decreases were 6 % per decade, 3 % per decade and 1 % per decade. Age is not a predictor of distension for the LPAs of the female cohort. No statistically significant differences were found in the slopes of these plots between males and females.

Figure 3 shows a plot of mean diastolic diameter against age in the MPA, RPA and LPA for both males and females. In the MPA, the rate of increase was 0.3 mm per decade for males and 0.1 mm per decade for females. In the RPA, the rate of increase was 1 mm per decade for males and 0.5 mm per decade for females. In the LPA, the increase was 0.9 mm per decade for males and 0.6 mm per decade for females. BSA is not a predictor of mean diastolic diameter for the MPAs of the female cohort.

Figure 4 shows the mean diastolic diameter of the MPA, RPA and LPA plotted against BSA. In each case, the mean diameter tends to increase with BSA, which is typically larger in males than in females.

## Discussion

With a view to the interpretation of both clinical findings and research studies, we present here a series of reference measurements of the pulmonary arteries made from appropriately located CMR cine acquisitions. As far as the authors are aware, this is only methodical study by CMR of the diameters, cross sectional areas and area changes of all three pulmonary arteries in a relatively large cohort of males and females, recruited prospectively to range across six decile age groups. We made our measurements using bSSFP cine images, which have the advantages of good blood-tissue contrast and high temporal resolution [4].

Several previous reports of pulmonary artery diameters have been based on CT or echocardiography, each of which have certain restrictions [1, 2, 8–12]. CT has the disadvantage of exposing patients to ionizing radiation, especially if acquired at more than one ECG gated phase of the cardiac cycle, and is generally considered unsuitable for studies of healthy volunteers. In the majority of such studies, measurements were made from planes oblique to or aligned with the artery in question, measuring the widest vessel diameter perpendicular to the longer apparent vessel axis, not necessarily in planes imaged or reconstructed to show the cross sectional area. Echocardiography can be suboptimal due to limited acoustic access to the PAs, which cannot necessarily be visualized in a cross-sectional view, so the cross sectional area can only be deduced from a single measured diameter in each vessel region [13].

Only a few previous CMR studies have included measurements of PA distension. Two early studies included relatively small groups of 17 and 9 healthy adult volunteers, respectively, [14, 15] In the latter study, distensibility was calculated using the formula: *(systolic area minus diastolic area) divided by systolic area, expressed as a percentage*. Use of the systolic rather than diastolic area as the denominator may in part have accounted for the relatively low values of about 25 % which they reported. Furthermore, the acquisition sequences they used differed from ours and might no longer be considered suitable, being gradient echo cine, [14] or static spin echo images acquired at end diastole and end systole during free breathing, and not, in the case of spin echo images, at the phase of peak systolic distension [15]. More recently, Shariat et al., [4] with a view to assessing differences of pulmonary arterial luminal diameter measured by different CMR approaches, studied 26 patients with repaired tetralogy of Fallot and pulmonary regurgitation and 26 patients who had undergone CMR for suspected arrhythmogenic right ventricular cardiomyopathy (ARVC). Both groups were relatively young, most being in the adolescent and teenage range, and they focused on diameters of the RPA. They found systolic bSSFP measurements to be reproducible between observers and comparable with those derived from non-cardiac gated 3D contrast enhanced magnetic resonance angiography, with the added advantage that the cine enables minimal diastolic as well as maximal systolic diameters to be measured, and without the need for contrast injection. In our study, decreasing measurements of distension of the PAs with age was as expected due to the known effects of the ageing process on vascular compliance. A study seeking to establish normal values for MPA, RPA and LPA diameters by contrast-enhanced magnetic resonance angiography in children and adolescents is not directly comparable with ours on account of the different methods and age range [16].

Our mean maximum systolic values were generally similar to those previously reported in adults from CT and echo studies, although usually slightly larger. This may reflect slight exaggeration of the apparent lumen size by the bright blood bSSFP technique together with partial volume averaging in the boundary pixels. As mean diastolic diameter measurements consistently gave more favorable R^2^ values than the systolic, they have a potential advantage as reference values for pulmonary artery size when investigating any dilatation that could result from pulmonary arterial hypertension, excess flow due to left to right shunting, a connective tissue disorder or post-stenotic dilatation downstream of a stenosis. Containment within the pericardium of the ascending aorta as well as the MPA may contribute to the relatively low systolic R^2^ values for MPA diameters and area relative to age or BSA, and may also account for the lack of MPA dilatation with age. There were slight differences of slope between the plots of male and female PA measurements of distension and mean diameter against both age and BSA, and we remain uncertain about their possible causes or significance.

In patients where pulmonary artery stenosis or hypoplasia is a concern, the maximum measurements made in systole are likely to be more relevant than those in diastole, systole being the period of forward flow.

### Limitations of the study

As our study did not include measurements of pulmonary artery pressures, it was only possible for us to report pulmonary artery distension, not distensibility. We have not attempted to quantify the inter-study or inter-observer reproducibility of the measurements made. The bSSFP methods used gave very clear delineation of vessel boundaries and were expected to entail only minor variability, as previously reported [4]. The branch pulmonary artery measurements were acquired from images transecting the vessels located on axial scout images without correction for any variation in cranio/caudal direction of the vessels. Any resulting angulation relative to the local vessel axis is likely to have been relatively small in the volunteers studied. However, possible obliquity could result in overestimation of both the larger dimension and the apparent cross sectional area. As the geometry of abnormal vessels can be variable, additional scout images aligned with each PA branch and orthogonal to the transaxial scouts would be advisable before transection of the vessel. The healthy volunteers imaged in this study may have had better compliance with the breath-holding requirements of CMR than some potential patients. Parallel imaging techniques were not used, but could be implemented either to reduce acquisition time in breathless patients or to improve spatial or temporal resolution, albeit at the expense of signal-to-noise ratio in the resulting images. In this study of healthy volunteers we acquired cine images with a temporal resolution of 46 ms. Although we considered this adequate, higher temporal resolution might be needed to enable more precise identification of the end systolic and end diastolic frames in patients with tachycardia. Although multivariate linear regression analysis of measurements in our cohort using age and BSA as input variables showed favourable R2 values, the ten subjects in each decile group for each gender were not sufficient to determine the 5th and 95th percentile limits with respect to BSA for individual deciles.

## Conclusions

We report normal reference values for measurements by CMR SSFP cine acquisitions of the main and branch pulmonary arteries, with documentation of changes with age and BSA.
